# Expanded genetic analysis of a PALB2 c.3113G>A mutation carrying multiple-case breast cancer family via exome sequencing

**DOI:** 10.1186/1897-4287-10-S2-A92

**Published:** 2012-04-12

**Authors:** ZL Teo, DJ Park, F Odefrey, F Hammet, T Nguyen-Dumont, H Tsimiklis, BJ Pope, A Lonie, I Winship, GG Giles, JL Hopper, MC Southey

**Affiliations:** 1 Genetic Epidemiology Laboratory, The University of Melbourne, Australia; 2 Victorian Life Sciences Computation Initiative, Australia; 3 Department of Medicine, The University of Melbourne, and Royal Melbourne Hospital, Australia; 4 Cancer Epidemiology Centre, The Cancer Council Victoria, Australia; 5 Australian Breast Cancer Family Study, Centre for Molecular Environmental Genetic and Analytical Epidemiology, School of Population Health, The University of Melbourne, Australia; 6 Centre for Molecular Environmental Genetic and Analytical Epidemiology, School of Population Health, The University of Melbourne, Australia

## 

*PALB2* is a breast cancer (BC) susceptibility gene. Its product is the binding partner of BRCA1 and BRCA2 and is involved in DNA repair. Studies of multiple-case BC families have reported that truncating mono-allelic *PALB2* mutations, on average, increase BC risk two to six fold.

Our recent population-based study estimated that carriers of *PALB2* c.3113G>A had a cumulative BC risk of carrying *PALB2* c.3113G>A to be 91% (95%CI=41-100) by age 70, comparable to 65% (95%CI=44-78) and 45% (95%CI=31-56) average risk estimates for *BRCA1* and *BRCA2* mutation carriers respectively. One of these pedigrees used for the above estimation, extensively screened but not found to carry a *BRCA1* or *BRCA2* mutation, exhibited ten diagnoses of BC, including five under 50 years of age, and a pedigree structure that does not allow for all cases to be explained by a single genetic factor. In view of such a high risk estimate and the observations of BC in non-*PALB2* mutation carriers in this and other pedigrees, we hypothesise that there may be other genetic risk factors segregating through this family.

Several genetic modifiers of BC risk have been verified in *BRCA1* and *BRCA2* mutation carriers. Although these genetic modifiers are associated with small changes in risk individually, collectively they may account for considerable risk modification for some women. Other moderate to highly penetrant genetic factors could also influence risk in *BRCA1* and *BRCA2* mutation carriers and non-carriers in BC families harbouring such genetic variants. Similarly, the presence of other genetic factors in the subject *PALB2* c.3113G>A pedigree of this study, could alter the risk for mutation carriers and/or convey BC risk to non-*PALB2* mutation carriers. Such additional factors could further explain the multiple cases of early onset BC and the high estimated risk of *PALB2* c.3113G>A in this family.

Whole exome capture followed by massively parallel sequencing was performed on four strategically selected affected females of this family. We have shortlisted 15 variants in this family predicted to be protein damaging by SIFT in genes with plausible relevance to cancer aetiology. Eight such variants are predicted to result in protein truncation or are variants predicted to have strong effects on splicing efficiency, one of which have been observed in key DNA repair genes. Six of the genes have roles in DNA repair, three in cell cycle checkpoint regulation, one controls telomerase activity and another has been reported to be a prostate cancer predisposition gene. Further analyses of these candidate BC predisposition genes will be presented.

